# Enhanced Stability of DNA Oligonucleotides with Partially Zwitterionic Backbone Structures in Biological Media †

**DOI:** 10.3390/molecules23112941

**Published:** 2018-11-10

**Authors:** Melissa Meng, Boris Schmidtgall, Christian Ducho

**Affiliations:** 1 Saarland University, Department of Pharmacy, Pharmaceutical and Medicinal Chemistry, Campus C2 3, 66123 Saarbrücken, Germany; melissa.meng@uni-saarland.de (M.M.); boris.schmidtgall@gmail.com (B.S.); 2 University of Paderborn, Department of Chemistry, Warburger Str. 100, 33098 Paderborn, Germany

**Keywords:** DNA, oligonucleotides, backbone modifications, nucleases, biological media

## Abstract

Deficient stability towards nuclease-mediated degradation is one of the most relevant tasks in the development of oligonucleotide-derived biomedical agents. This hurdle can be overcome through modifications to the native oligonucleotide backbone structure, with the goal of simultaneously retaining the unique hybridization properties of nucleic acids. The nucleosyl amino acid (NAA)-modification is a recently introduced artificial cationic backbone linkage. Partially zwitterionic NAA-modified oligonucleotides had previously shown hybridization with DNA strands with retained base-pairing fidelity. In this study, we report the significantly enhanced stability of NAA-modified oligonucleotides towards 3′- and 5′-exonuclease-mediated degradation as well as in complex biological media such as human plasma and whole cell lysate. This demonstrates the potential versatility of the NAA-motif as a backbone modification for the development of biomedically active oligonucleotide analogues.

## 1. Introduction

As they efficiently modulate biological processes, oligonucleotides hold immense potential to serve as scaffolds for novel pharmaceutical agents. Due to their sequence-specific hybridization to DNA duplexes or single-stranded mRNA, single-stranded exogenous oligonucleotides can display antigene or antisense activity, respectively. In contrast, exogenous double-stranded RNA (siRNA) can regulate gene expression via the RNA interference mechanism [1]. However, the high polarity of native nucleic acid structures is a major disadvantage with these approaches. The oligoanionic phosphate diester-linked backbone significantly hampers cellular uptake and accounts for an overall poor pharmacokinetic profile. Furthermore, the phosphate-sugar backbone is prone to nuclease-mediated cleavage, thus further preventing applications in vivo.

As a consequence, numerous chemical modifications to the nucleic acid backbone have been studied with the aim of altering its polarity and sensitivity to enzymatic degradation [2,3]. The structural variety of such synthetic artificial linkages ranges from minor alterations to a complete replacement of the native internucleotide connection. In this context, phosphorothioates are one of the earliest and most well-established backbone variations [4]. This rather minor modification prevents nuclease-mediated cleavage while preserving the overall architecture of the native DNA structure. While phosphorothioates conserve the native negative charge pattern of the backbone, several artificial nucleic acid mimics contain electroneutral internucleotide linkages, thus aiming for enhanced lipophilicity and, therefore, for improved penetration of cellular membranes. For instance, non-natural electroneutral modifications such as triazoles [5,6,7,8,9], amides [10,11,12,13,14,15], phosphate triesters [16] and sulfones [17] have been developed. Another electroneutral congener that displays only a remote resemblance to the native DNA backbone is the amide-based peptide nucleic acid (PNA) [18,19]. PNA exhibits high fidelity in sequence-specific hybridization with native nucleic acid strands. However, fully electroneutral oligonucleotide analogues tend to form aggregates and suffer from low water solubility, thus hampering their potential biomedical application. 

An alternative approach to modifying the oligoanionic architecture of nucleic acid strands is the introduction of positive charges into the oligomer, which enables a partial ‘masking’ of its anionic motifs and may lead to zwitterionic nucleic acid analogues. In many such approaches, the oligoanionic phosphate diester backbone was retained, while the positively charged moieties were attached either to the nucleobase or the 2′-hydroxy group (in RNA) [20,21,22,23]. The resultant oligonucleotide analogues display a zwitterionic character but also densely charged structures, as the positively charged moiety is just an additional feature.

One approach to overcoming such limitations is the introduction of positively charged units as a site-specific replacement of the native phosphate diester internucleotide linkages. This may furnish partially or fully zwitterionic or even fully cationic oligonucleotide analogues, dependent on the number of artificial cationic linkages in a sequence. The altered properties of an accordingly modified nucleic acid strand might potentially be advantageous for biomedical applications with respect to nuclease stability and cellular uptake. Remarkably, this strategy has received only limited attention so far [24,25]. Undertaking pioneering work in this field, Letsinger and co-workers have introduced the conformationally flexible alkylphosphoramidate linkage [26], which later found use in several related systems [25]. Bruice and co-workers have developed conformationally rigid cationic linkages, i.e., guanidine [27,28,29] and *S*-methylthiourea units [30,31].

**Figure 1:**
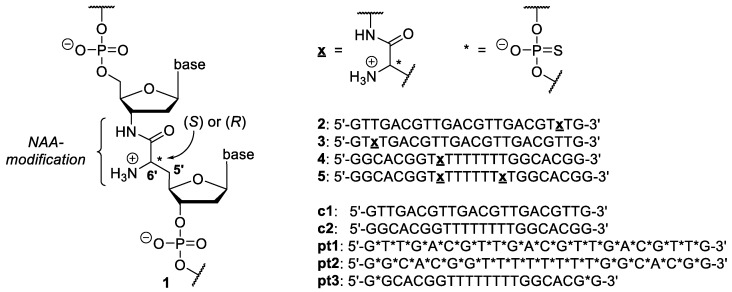
Principle structure **1** of an NAA-modified DNA oligonucleotide with a partially zwitterionic backbone; NAA-modified DNA oligonucleotides **2**–**5** investigated in this study with unmodified controls **c1** and **c2** as well as phosphorothioate-modified controls **pt1**–**pt3** (the linkages not highlighted are phosphate diesters; all sequences are 2′-deoxy).

We have introduced a novel cationic internucleotide linkage with intermediate conformational flexibility named the ‘nucleosyl amino acid (NAA)-modification’. This artificial backbone motif was proposed to be conformationally advantageous relative to Letsinger’s and Bruice’s systems [32]. Therefore, it was initially employed to prepare partially zwitterionic oligonucleotides of type **1** (Figure 1) [32,33]. In a recent study, we have also reported fully cationic oligonucleotide analogues with backbones solely comprising NAA internucleotide linkages [34]. The NAA-modification was originally inspired by the ‘high-carbon’ nucleoside core structure of muraymycin nucleoside antibiotics and structurally simplified analogues thereof [32,35,36,37,38]. Thus, the stereochemical configuration in the 6′-position determines the spatial orientation of the positive charge (Figure 1).

In the context of partially zwitterionic NAA-modified oligonucleotides of type **1**, we have synthesized a series of such oligonucleotides and studied their hybridization with DNA and RNA counterstrands, both with full base complementarity and containing single-base mismatches [32]. The most important results for partially zwitterionic NAA-modified DNA oligonucleotides were as follows: (i) they hybridized with complementary unmodified DNA and RNA counterstrands to form duplexes; (ii) these duplexes were moderately destabilized (with DNA) and fairly significantly destabilized (with RNA), relative to the corresponding native duplexes; (iii) the spatial orientation of the positive charge (6′*S*/6′*R*) only had a minor influence on duplex stability, with a tendency for the (6′*R*)-configuration to furnish slightly more stable duplexes with native DNA or RNA counterstrands; (iv) base-pairing fidelity was retained, i.e., a single-base mismatch in the counterstrand led to significant destabilization of the duplex; (v) for the DNA-DNA duplexes, no significant distortion of the helical structure was found using CD spectroscopy [32]. Hence, it was concluded that fundamental chemical characteristics of nucleic acids are retained in NAA-modified DNA oligonucleotides. This qualifies the NAA-modification as an attractive addition to the existing ‘toolbox’ of nucleic acid backbone modifications. Remarkably, the recently reported fully cationic NAA-derived oligonucleotide analogues showed impaired base-pairing fidelity [34], thus indicating that the NAA-modification might only be suitable for (partially) zwitterionic backbone structures.

In order to further establish the NAA-modification as a versatile structural alteration of the nucleic acid backbone, detailed information on its properties in biological media are required. This particularly concerns the stability of NAA-modified oligonucleotides towards nuclease-mediated degradation. Up to this point, only very limited insights into the potential overall stabilization of the oligonucleotide backbone due to the presence of cationic linkages have been available. As previously reported by Bruice, ‘chimeric’ zwitterionic oligonucleotides with an artificial cationic linkage at the 3′-end (either with a guanidinium [28] or *S*-methylthiourea [31] moiety), were completely stable towards degradation by 3′→5′-exonucleases. However, the influence of 5′→3′-nucleases on such partially zwitterionic structures has not been studied yet. Furthermore, no investigations regarding the influence of some positive charges in the oligonucleotide backbone on its stability in more complex biological media (plasma, cell lysates) are available.

Hence, in this work, we report on the in vitro stability of some selected zwitterionic NAA-modified DNA oligonucleotides in nuclease-containing buffer as well as in complex biological media such as human plasma and cell lysates. Most likely, the NAA-modification itself will not be prone to enzymatic cleavage. It needs to be elucidated, however, whether this artificial backbone motif simply serves as a ‘stopper’ in nuclease-mediated degradation of the backbone or if it might exert a general stabilizing effect. In this context, the term ‘general stabilizing effect’ means that the sequence may be degraded until the NAA-modification is reached, but that the overall half-life of the oligonucleotide is increased relative to an unmodified native congener. 

For the according studies, we have selected a set of four previously reported NAA-modified DNA oligonucleotides **2**–**5** (Figure 1) [32]. In sequences **2** and **3**, the NAA-modification is placed in close proximity to the 5′- and 3′-ends, respectively, with one adjacent phosphate diester linkage at the terminus. In oligonucleotides **4** and **5**, one or two NAA-modifications are placed in an oligo-T internal segment of the sequence. With respect to their slightly superior hybridization properties, oligomers with (6′*R*)-configuration in the NAA unit were chosen, except where indicated. Furthermore, oligonucleotides **c1** and **c2** served as unmodified native controls for **2**/**3** and **4**/**5**, respectively. Phosphorothioate-linked oligonucleotides **pt1**–**pt3** were employed as stable, non-degradable additional controls for **2**/**3** (**pt1**) and **4**/**5** (**pt2** and **pt3**), respectively (Figure 1). 

## 2. Results

### 2.1. Stability of NAA-modified Oligonucleotides towards Nuclease-mediated Cleavage 

First, we investigated the in vitro stability of **2**–**5** towards nuclease-mediated degradation using nuclease-containing buffer. Based on literature precedent [39,40,41], two exonucleases commonly employed to test the stability of DNA oligonucleotides were chosen: the 3′→5′ exonuclease snake venom phosphodiesterase (SVP) from *Crotalus adamanteus* and the 5′→3′ exonuclease bovine spleen phosphodiesterase (BSP), respectively. Endonucleases only target duplex structures and therefore play a less significant role with respect to potential biomedical applications of single-stranded oligonucleotide analogues. Consequently, they were not included in the assay. After incubation with one of the two exonucleases, the assay mixtures were analyzed by denaturing polyacrylamide gel electrophoresis (PAGE) using urea-containing gels. Concentrations of the oligonucleotides were chosen with respect to their UV detection limits, and the amounts of enzyme (0.4 mU SVP, 30 mU BSP) were adjusted so that unmodified oligonucleotides were completely degraded within less than two hours at 37 °C.

**Figure 2:**
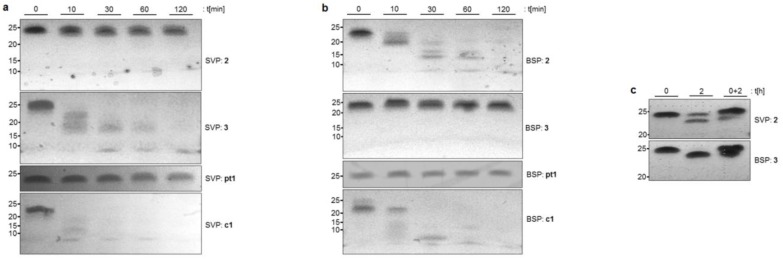
(**a,b**) Nuclease assays with NAA-modified oligonucleotides **2** and **3**, controls **c1** and **pt1** and exonucleases SVP (3′→5′, **a**) and BSP (5′→3′, **b**). (**c**) Sequencing gel analysis of the assays with oligonucleotide **2** and SVP, and **3** and BSP, respectively; left lane: assay mixture after 0 h incubation time, middle lane: assay mixture after 2 h incubation time, right lane: mixture of assay mixtures after 0 h and 2 h incubation times. In the interest of clarity, the oligonucleotide ladder indicating the lengths of the oligomers is not displayed and has been replaced with labels on the left side of the gels.

Using these conditions, native control **c1** and phosphorothioate-modified control **pt1** were incubated with either SVP or BSP and analyzed (Figure 2). As anticipated, **c1** was degraded rapidly, whereas the fully phosphorothioate-modified oligomer **pt1** remained stable over the complete incubation period. When NAA-modified oligomer **2** (with (6′*R*)-configuration in the NAA unit) was tested with the SVP 3′→5′ exonuclease, it showed excellent stability (Figure 2a). In contrast, when the same oligonucleotide was subjected to BSP 5′→3′ exonuclease activity, it underwent rather rapid degradation (Figure 2b). Remarkably, this nuclease-mediated cleavage appeared to be slowed down relative to the degradation of native control **c1**. The results were inverted when NAA-modified oligomer **3** was tested under identical conditions. It showed degradation with SVP (though again, apparently not as rapid as native control **c1**, Figure 2a), but excellent stability with BSP (Figure 2b). However, for both NAA-modified oligomers **2** and **3**, cleavage of the terminal nucleotide located in immediate proximity to the NAA-modification could be verified (**2** with SVP and **3** with BSP) using a higher resolution sequencing gel (Figure 2c).

**Figure 3:**
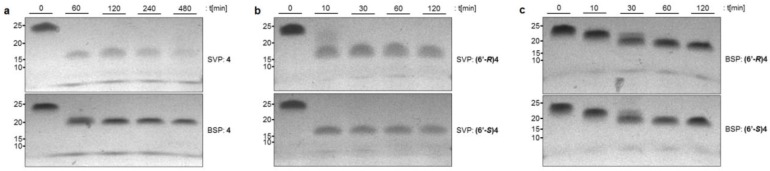
(**a**) Nuclease assays with NAA-modified oligonucleotide **4** and either SVP or BSP exonuclease over a time course of eight hours. (**b**,**c**) Nuclease assays with diastereomers of **4** differing in the configuration of the NAA 6′-stereocenter (6′*S*/6′*R*) using SVP (**b**) or BSP (**c**). In the interest of clarity, the oligonucleotide ladder indicating the lengths of the oligomers is not displayed and has been replaced with labels on the left side of the gels.

In a next step, we then studied the nuclease stability of oligonucleotide **4** with an internal NAA-modification. In this context, we also aimed to study longer incubation periods. Thus, oligomer **4** was treated with either SVP or BSP over a total time course of eight hours. In both assays, degradation of the termini, but not beyond the point of modification, was detected (Figure 3a). Another aspect of interest was the influence of the configuration of the NAA 6′-stereocenter on nuclease stability. Therefore, both the (6′*R*)- and the (6′*S*)-configured versions of **4** were incubated with SVP and BSP, respectively, over two hours (Figure 3b,c). However, no notable difference in nuclease stability was observed, indicating that the stereochemical configuration of the NAA-modification was not relevant to its effect on stabilization towards nucleases. It should be noted that the presence of the NAA-modification slowed down BSP-mediated degradation more efficiently.

### 2.2. Stability of NAA-modified Oligonucleotides in Complex Biological Media

**Figure 4:**
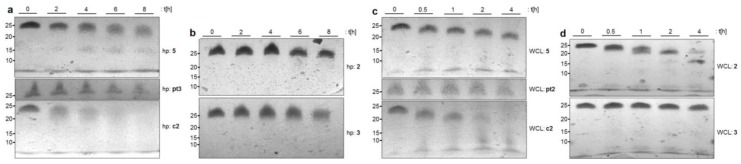
(**a**,**b**) Influence of human plasma (hp) on NAA-modified oligonucleotides **2**, **3** (**b**) and **5** (**a**) with controls **c2** and **pt3**. (**c**,**d**) Influence of whole cell lysate (WCL) on NAA-modified oligonucleotides **2**, **3** (**d**) and **5** (**c**) with controls **c2** and **pt2**. In the interest of clarity, the oligonucleotide ladder indicating the lengths of the oligomers is not displayed and has been replaced with labels on the left side of the gels.

The aforementioned nuclease assays are versatile but simplified model systems to elucidate the biochemical stability of NAA-modified DNA oligonucleotides. Consequently, we subsequently studied their properties in vitro in more complex biological media (Figure 4). First, the stabilities in human plasma were investigated using a slightly adjusted protocol (as the addition of organic solvent after incubation led to the precipitation and agglomeration of plasma components). With unmodified control oligonucleotide **c2**, the results were as expected as it underwent full cleavage. Remarkably, phosphorothioate-modified control oligonucleotide **pt2** gave no detectable bands in the ureaPAGE gel. This phenomenon might correlate with the pronounced plasma protein binding of phosphorothioates [42] and was circumvented using **pt3** as a control instead, which only had two terminal phosphorothioate modifications to mediate exonuclease stability. As anticipated, **pt3** was found to be reasonably stable over nearly the whole eight-hour time course of incubation (Figure 4a). The odd shape of the bands in the gel probably resulted from some remaining unspecific binding to plasma proteins and the slow dissociation of **pt3** from these complexes. The influence of more complex biological media on DNA analogues with partially zwitterionic backbones had not been studied before. Oligomer **5** was chosen to enable a sequential degradation from the 3′- as well as from the 5′-end. It was envisioned that further insights would be obtained into the potential general stabilizing effect of the NAA-linkage. For NAA-modified oligonucleotide **5**, some slight initial degradation in human plasma was observed, which resulted in the accumulation of a shorter fragment over the full time course (Figure 4a). Overall, the partial degradation of **5** in plasma was significantly slower than the full cleavage of unmodified control **c2**.

We then performed additional studies on human plasma with oligomers **2** and **3,** which are modified close to the termini. Both sequences showed high stability over a course of eight hours when compared to **c2** (Figure 4b). Oligonucleotide **2** seems to undergo some initial cleavage within the first 4 h. However, the sharp bands appearing at time points 6 and 8 h, respectively, indicate no further decomposition and therefore stabilization of the sequence. For 5′-modified congener **3**, a degradation process similar to **5** was observed.

We also examined stabilities in whole cell lysate (human U937 lymphoma cell line). This assay was performed in a similar manner as the assays for nuclease stabilities (vide supra). In this case, control **pt2** gave more defined bands than in the plasma assays, and therefore, its anticipated stability in cell lysate could be proven. In contrast, native control **c2** was rapidly degraded (Figure 4c). However, incubation of NAA-modified oligomer **5** with whole cell lysate resulted in considerably slower cleavage. Remarkably, the degradation process had not even reached the modified backbone segment by the time native control **c2** was already completely decomposed (Figure 4c). Further assays were performed using oligomers **2** and **3**. Relative to control **c2**, a single NAA-linkage positioned adjacent to the 3′-end (as in **2**) slightly slowed down degradation, while the congener with the modification close to the 5′-position (i.e., **3**) showed high stability over the complete period of incubation (Figure 4d).

## 3. Discussion

Overall, the assays performed with SVP and BSP nucleases (Figure 2) strongly indicate that, as expected, the NAA-modification was not hydrolyzed by the nucleases. For instance, NAA-modified oligomer **3** (with the modification close to the 5′-end) showed 3′→5′ degradation with SVP (though apparently not as rapid as native control **c1**, Figure 2a), but excellent stability towards 5′→3′ cleavage mediated by BSP (Figure 2b). It can also be derived that the presence of the NAA-modification apparently slows down nuclease-mediated cleavage of adjacent phosphate diester linkages (**2** with SVP, **3** with BSP). However, for both NAA-modified oligomers **2** and **3**, cleavage of the terminal nucleotide located in immediate proximity to the NAA-modification could be verified (**2** with SVP, **3** with BSP, Figure 2c). This is in some contrast to the findings of Bruice, who reported no cleavage of the unmodified nucleotide at the 3′-terminus of partially zwitterionic backbones containing his modifications [28,31]. On the other hand, the intact oligonucleotide was still found alongside the degradation product after two hours incubation of oligomer **2** (Figure 2c). This further confirms that the nuclease-mediated hydrolysis of phosphate diester linkages adjacent to NAA-modified sites is decelerated relative to native backbone structures. The observation that a single NAA-modification in a distant position also furnished a slight stabilization of the oligonucleotides (**3** with SVP, **2** with BSP, Figure 2a,b) hinted towards a moderate general stabilizing effect of the cationic modification in spite of its remote position. This might be due to either: (i) a potentially reduced binding affinity to nucleases as a consequence of the zwitterionic segment of the backbone; and/or (ii) the formation of transient folded structures of the oligomer as a result of intramolecular eletrostatic attraction.

For the internally modified oligomer **4**, the presence of the NAA-modification slowed down BSP-mediated degradation more efficiently relative to the (more rapid) SVP-catalyzed cleavage (Figure 3). This observation is in line with BSP being a 5′→3′ exonuclease as the NAA-modification is placed closer to the 5′-end in oligomer **4**. It might also indicate though that BSP is generally more sensitive to an altered charge pattern in the oligonucleotide backbone than SVP.

When NAA-modified oligonucleotide **5** was incubated in human plasma, some slight initial degradation was observed, which resulted in the accumulation of a shorter fragment over the full time course (Figure 4a). This indicated that, most likely, no degradation beyond the position of the NAA-modification occurred. Overall, the partial degradation of **5** in plasma was significantly slower than the full cleavage of unmodified control **c2**. For the incubation of 5′-modified congener **3** in human plasma, a degradation process similar to **5** was observed, while 3′-modified oligomer **2** showed stabilization of the sequence after initial cleavage. One can therefore conclude that a single NAA-modification at the 3′-end (as in **2**) sufficiently stabilized the remaining phosphate diester backbone and that the NAA-modification overall furnishes a general stabilizing effect in human plasma.

For incubations in human cell lysate, it was observed that the degradation process of doubly NAA-modified oligonucleotide **5** had not even reached the modified backbone segment by the time native control **c2** was already completely decomposed (Figure 4c). As for the assays with exonucleases (vide supra), the NAA-linkage seems to exert a general stabilizing effect. This was further verified by the results from the according assays with oligomers **2** and **3**. Relative to control **c2**, a single NAA-linkage adjacent to the 3′-end (as in **2**) slightly slowed down degradation, while the congener with the modification close to the 5′-position (i.e., **3**) showed high stability (Figure 4d). These results also indicate that 5′-exonuclease activity appears to be significantly higher than 3′-exonuclease activity in the investigated U937 human lymphoma cell line.

## 4. Materials and Methods

### 4.1. Synthesis of NAA-Modified Oligonucleotides

The synthesis of NAA-modified oligonucleotides **2**-**5** was performed as reported earlier [32]. Briefly, a ‘dimeric’ T-T phosphoramidite building block containing the protected NAA-linkage was employed in solid phase-supported automated DNA synthesis. After cleavage from the resin and concomitant deprotection under standard conditions, the resultant NAA-modified oligonucleotides were purified using gel elelctrophoresis and precipitation [32,33].

### 4.2. Stability Assay with 3′-exonuclease

The stability of oligonucleotides against 3′-exonuclease-mediated degradation was determined using snake venom phosphodiesterase from *Crotalus adamanteus* venom (vial of ≥ 0.40 units, purified, Sigma-Aldrich, Saint Louis, MO, USA). The enzyme was dissolved in water according to the manufacturer’s protocol. The oligonucleotide (1.4 nmol, 7 μL of 200 nM stock solution in water) was mixed with glycine buffer (200 mM glycine, 15 mM MgCl_2_, pH 9) to a volume of 92 μL and then cooled to 0 °C. Snake venom phosphodiesterase (0.4 mU, 8 μL of 0.05 mU/μL stock solution) was added. The resultant assay mixture was incubated at 37 °C for a total of 120 and 480 min, respectively. Samples were taken at the time points indicated in the Figures. The enzymatic reaction was quenched by mixing the sample (17.9 μL assay mixture, corresponding to 0.25 nmol oligomer) with MeCN (17.9 μL). The resultant mixture was dried in vacuo, dissolved in 7 μL 0.5x TBE-running buffer (10x TBE-running buffer: 890 mM Tris, 890 mM boric acid, 20 mM EDTA, pH 8.3) and mixed with 4 μL loading buffer (90% formamide, 10% glycerol, bromophenol blue). Analysis of the samples was performed on a ureaPAGE gel (4.75 g urea (7 M), 5.63 mL Rotiphorese™ (Carl Roth, Karlsruhe, Germany), 1.125 mL 10x TBE-running buffer, 11.25 mL water, 8.75 μL TEMED, 62.5 μL APS (10% in water)) run at room temperature for 2 h. Bands were detected using UV (254 nm) after placing the gel on a plastic foil-covered TLC plate (aluminum plate precoated with silica gel 60 F_254_ (VWR)).

### 4.3. Stability Assay with 5′-exonuclease

The stability of oligonucleotides against 5′-exonuclease-mediated degradation was determined using phosphodiesterase II, bovine spleen phosphodiesterase (vial of 10 units, purified, Sigma-Aldrich). The enzyme was dissolved in water according to the manufacturer’s protocol. The assay was performed as described for 3′-exonuclease (vide supra), with the following differences: the oligonucleotide (1.4 nmol, 7 μL of 200 nM stock solution in water) was mixed with acetate buffer (100 mM ammonium acetate, 1 mM EDTA, 1 mM TWEEN 80, pH 6-7) to a volume of 97 μL and cooled down to 0 °C. Bovine spleen phosphodiesterase (30 mU, 3 μL of 10 mU/μL stock solution) was then added.

### 4.4. Stability Assay with Human Plasma

The stability of oligonucleotides against degradation in plasma was determined using pooled human plasma (BIOTREND Chemikalien GmbH, Cologne, Germany). The oligonucleotide (1.4 nmol, 7 μL of 200 nM stock solution in water) was mixed with 38 μL undiluted human plasma and cooled to 0 °C. The resultant assay mixture was incubated at 37 °C for a total of 480 min. Samples were taken at the time points indicated in the Figures. The degradation process was quenched by mixing the sample (8 μL assay mixture, corresponding to 0.25 nmol oligomer) with stopping solution (9 M urea, 15% glycerol, 1:1, 8 μL) and storing it on ice. An analysis of the resultant mixtures was performed on a ureaPAGE gel as described above.

### 4.5. Stability Assay with Whole Cell Lysate

The stability of oligonucleotides against degradation in whole cell lysate was determined using self-prepared lysate of the U937 cell line. U937 cells (Sigma-Aldrich) were cultured according to the manufacturer’s protocol. For cell lysis, a general lysis buffer (50 mM Tris-HCl pH 7.5, 100 mM NaCl, 5% glycerol) was used. Immediately prior to lysis, 1 mM dithiothreitol (DTT) and 250 mM phenylmethylsulfonyl fluoride (PMSF) were added to the lysis buffer. Subsequently, the pellet (cell count before harvesting ~150,000,000 cells) of a freshly harvested U937 cell culture was mixed with 40 mL of general lysis buffer and ultra-sonicated (5 cycles, 15 pulses, 20%) with the cell suspension being stored on ice. Centrifugation (13 000 rpm, 30 min, 4 °C) and collecting the supernatant furnished the final whole cell lysate. The oligonucleotide (1.4 nmol, 7 μL of 200 nM stock solution in water) was mixed with 93 μL undiluted whole cell lysate. The resultant assay mixture was incubated at 37 °C for a total of 240 min. Samples were taken at the time points indicated in the Figures. The degradation process was quenched by mixing the sample (17.9 μL assay mixture, corresponding to 0.25 nmol oligomer) with MeCN (17.9 μL). The resultant mixture was dried in vacuo, dissolved in 7 μL 0.5x TBE-running buffer (vide supra for 10x TBE-running buffer) and mixed with 4 μL loading buffer (vide supra). Analysis of the samples was performed on a ureaPAGE gel as described above.

## 5. Conclusions

In summary, we have reported the first systematic study on the effect of an artificial cationic internucleotide linkage on the stability of corresponding partially zwitterionic oligonucleotides. We have investigated the degradation of NAA-modified zwitterionic oligonucleotides in different media, i.e., nuclease-containing buffer, human plasma, and human whole cell lysate. Overall, we have demonstrated the enhanced stability of such oligonucleotide analogues. No indication was found for a cleavage or excision of the NAA-modified site even in complex biological media (i.e., plasma and cell lysate). Most NAA-modified oligonucleotides investigated herein showed a remarkable general stabilization both in human plasma and in cell lysate, often even when the cationic NAA-modification was not in close proximity to the termini of the sequence. This might be due to a generally weakened binding of partially zwitterionic oligonucleotides to degrading enzymes such as exonucleases, most likely as a result of the altered charge pattern in the backbone. Alternatively, nuclease-mediated degradation might be slower due to the formation of transient folded structures of the zwitterionic oligomers as a result of intramolecular electrostatic attraction. As a consequence, placing only a few cationic NAA-linkages into an otherwise unmodified sequence should be sufficient to significantly enhance its stability in complex biological media. These beneficial properties complement the other favorable characteristics of NAA-modified zwitterionic oligonucleotides, such as their ability to form reasonably stable duplexes and their retained base-pairing fidelity. Overall, our results therefore confirm that the cationic NAA-linkage appears to be a potentially useful addition to the existing ‘toolbox’ of artificial backbone linkages for the development of oligonucleotide-based therapeutics or diagnostics.
